# Diffusion Barriers Constrain Receptors at Synapses

**DOI:** 10.1371/journal.pone.0043032

**Published:** 2012-08-13

**Authors:** Marianne Renner, Claude Schweizer, Hiroko Bannai, Antoine Triller, Sabine Lévi

**Affiliations:** Institut de Biologie de l’Ecole Normale Supérieure (IBENS), Institut National de la Santé et de la Recherche Médicale U1024, Centre National de la Recherche Scientifique UMR8197, Paris, France; The Research Center of Neurobiology-Neurophysiology of Marseille, France

## Abstract

The flux of neurotransmitter receptors in and out of synapses depends on receptor interaction with scaffolding molecules. However, the crowd of transmembrane proteins and the rich cytoskeletal environment may constitute obstacles to the diffusion of receptors within the synapse. To address this question, we studied the membrane diffusion of the γ-aminobutyric acid type A receptor (GABA_A_R) subunits clustered (γ2) or not (α5) at inhibitory synapses in rat hippocampal dissociated neurons. Relative to the extrasynaptic region, γ2 and α5 showed reduced diffusion and increased confinement at both inhibitory and excitatory synapses but they dwelled for a short time at excitatory synapses. In contrast, γ2 was ∼3-fold more confined and dwelled ∼3-fold longer in inhibitory synapses than α5, indicating faster synaptic escape of α5. Furthermore, using a gephyrin dominant-negative approach, we showed that the increased residency time of γ2 at inhibitory synapses was due to receptor-scaffold interactions. As shown for GABA_A_R, the excitatory glutamate receptor 2 subunit (GluA2) of the α-amino-3-hydroxy-5-methyl-4-isoxazolepropionic acid receptor (AMPAR) had lower mobility in both excitatory and inhibitory synapses but a higher residency time at excitatory synapses. Therefore barriers impose significant diffusion constraints onto receptors at synapses where they accumulate or not. Our data further reveal that the confinement and the dwell time but not the diffusion coefficient report on the synapse specific sorting, trapping and accumulation of receptors.

## Introduction

Concentration of neurotransmitter receptors in the postsynaptic membrane critically determines the efficacy of fast neurotransmission. Lateral diffusion in and outside synapses plays a key role in the regulation of receptor number at synapses [1], [2], [3]. Receptors continuously exchange between synaptic and extrasynaptic membranes. They display rapid free Brownian diffusion in extrasynaptic membrane and are slowed down and confined (restricted in space) at synapses. The restricted motion at synapses result from transient interactions of receptors with scaffolding molecules directly or indirectly bound to the cytoskeleton, a phenomenon also referred as the “diffusion-capture” mechanism (recently reviewed by [2]). Besides, the presence of obstacles in the synapse may also reduce the mobility of receptors. Obstacles to diffusion are created by a crowd of transmembrane proteins immobilized at the synapse through binding to the cytoskeleton [4]. These proteins include the receptors themselves as well as adhesion molecules that connect pre- and post- synaptic membranes such as the neurexin-neuroligin complex [5] and synaptic cell adhesion molecules with homophilic interactions (SynCam [6], Sidekicks [7] and cadherins [8], [9]). Also, cadherins by interacting with β-catenins can organize nanometers sized subdomains around the synapse [10]. The cytoskeletal fences constituted by the high density of cytoplasmic cytoskeletal elements present in the postsynaptic density are another source of obstacles to the diffusion of receptors. The membrane-associated portion of cytoskeletal proteins forms a corral from which transmembrane proteins can only escape by hop diffusion or by passing through gaps when the cytoskeleton is discontinuous (refs. in [11]). Last, receptor diffusion at synapses may be limited by their transient association with specialized lipid microdomains, the so-called “lipid rafts” [12], [13].

Although it has been demonstrated that obstacles alter the diffusion of lipids at synapses [14], this question has not been addressed for neurotransmitter receptors. Here we used quantum dot (QD)-based single particle tracking (SPT) as well as fluorescence recovery after photobleaching (FRAP) to compare diffusion behavior of GABA_A_Rs and AMPARs at excitatory and inhibitory synapses. Our main results showed that GABA_A_R and AMPAR diffusion is significantly reduced in synapses whatever the neurotransmitter contained in the presynaptic element. This suggests the lateral diffusion of receptors at mismatched synapses is hindered by the presence of diffusion barriers such as pickets and fences. Although the diffusion coefficients were similar at matching and mismatched PSDs, the explored area and dwell time of reflected receptor trapping at matching PSDs. Moreover, interfering with the clustering of the main inhibitory scaffolding protein gephyrin revealed that the receptor-scaffold interaction was responsible for the increased confinement and dwell time of GABA_A_R at inhibitory synapses, although no noticeable changes were observed in the diffusion coefficients. Thus, the explored area and dwell time, but not diffusion coefficient, are correlated with the synaptic sorting, trapping and concentration of receptors.

## Results

### Diffusion of the GABA_A_Rs γ2 and α5 Subunits at Inhibitory Synapses

**Figure 1:**
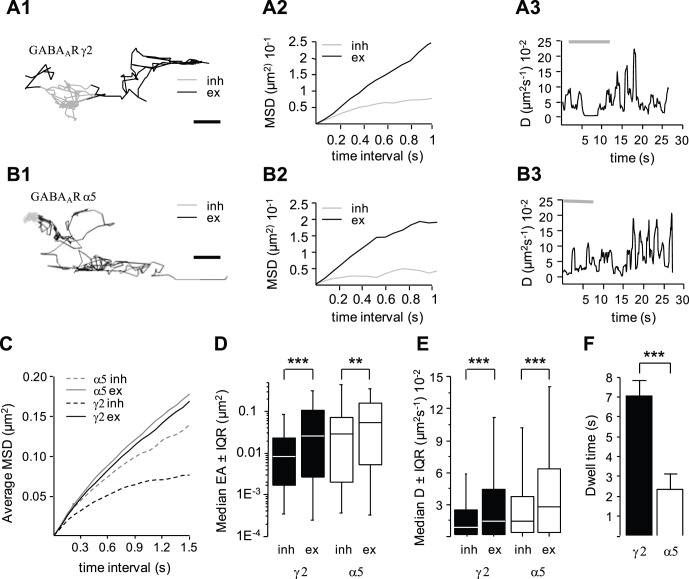
Membrane dynamics of the GABA_A_R γ2 and α5 subunits in relation with inhibitory synapses. (A, B) Examples of individual trajectories of QDs coupled to γ2 (A1) and α5 (B1) outside (black) or inside (grey) inhibitory synapses and their corresponding MSD (A2, B2) and instantaneous diffusion coefficients as a function of time (A3,B3). Scale, 0.5 µm (A1) and 1 µm (B1). Note the reduced diffusion and increased confinement of γ2 and α5 in inhibitory synapses *vs*. extrasynaptic membrane. (C–E) Diffusion behavior of receptor populations analyzed from pooled QD trajectories. Averaged MSD *vs*. time function (C), explored surface area (D, (median EA ±25%–75% Interquartile Range IQR; KS test ***p<10^−3^, **p<10^−2^), diffusion coefficient (E, median D ±25%–75% IQR; KS test ***p<10^−3^) of γ2 (black) and α5 (white) trajectories in inhibitory synapses (inh) and in extrasynaptic compartments (ex). (F) Dwell time (mean ± SEM) of the indicated receptors at inhibitory synapses (t-test, ***p<10^−3^).

GABA_A_Rs diffuse laterally on the neuronal plasma membrane and rapidly exchange between extrasynaptic and synaptic loci [15], [16], [17], [18], [19], [20], [21]. Using QD-based SPT [22], [23], we analyzed the mobility of the GABA_A_R γ2 subunit enriched at inhibitory postsynaptic site [24], [25], [26] and of the GABA_A_R α5 subunit found almost exclusively at extrasynaptic sites [27], [28], [29], [30]. The surface endogenous GABA_A_R γ2 and α5 subunits were labeled with specific antibodies directed against their extracellular N-terminus regions, subsequently labeled with specific intermediate biotinylated Fab fragments and streptavidin-coated QD (see Materials and Methods). The cell surface exploration of γ2 and α5 was visualized on trajectories reconstructed from 38.4 s recording sequences (e.g. Fig. 1A). To identify inhibitory synapses in live cells, neurons were transfected with a venus-gephyrin construct as done before [31], [32]. Extrasynaptic *vs*. synaptic trajectories were segregated by comparison with merge fluorescence images of venus-gephyrin and FM 4–64. Trajectories were at inhibitory synapses when overlapping with venus-gephyrin and FM 4–64 punctae (e.g. grey in Fig. 1A1, 1B1), or extrasynaptic for trajectories two pixels (440 nm) away (e.g. black in Fig. 1A1, 1B1) [22]. As exemplified in Fig. 1A1 for γ2, the surface exploration of the same QD was restricted to a smaller area at the synaptic *vs*. extrasynaptic compartment. This was also visible on the mean-square displacement function (MSD) versus time relation which showed a steeper slope in the extrasynaptic compartment (Fig. 1A2, same trajectory as in Fig. 1A1). Furthermore, the same QD had significantly lower mobility in synaptic *vs*. extrasynaptic compartment as seen on the instantaneous diffusion coefficient Dinst (Fig. 1A3). Quantitative analysis performed on a whole population of trajectories confirmed the reduced surface exploration of γ2 at inhibitory synapses (10 cultures; Kolmogorov-Smirnov KS test, p<10^−3^; Fig. 1C–D; Table 1) and the reduced lateral diffusion (KS test, p<10^−3^; Fig. 1E; Table 1) of γ2 at inhibitory synapses. These results are in agreement with earlier SPT work [18], [19], [20], [21] and are coherent with a capture of γ2 by the inhibitory scaffold. We then characterized the diffusion properties of α5. Despite their preferential non synaptic localization, α5-containing GABA_A_R also displayed reduced mobility at synapses (Fig. 1B1–3). Both the explored area (Fig. 1C–D and Table 1; 6 cultures; KS test, p = 0.002) and diffusion coefficient (Fig. 1E and Table 1; KS test, p<10^−3^) were reduced at inhibitory synapses vs. the extrasynaptic region. Therefore, GABA_A_Rs clustered (γ2) or not (α5) at inhibitory synapses showed a drop in their diffusion coefficient and an increase in their confinement at inhibitory synapses. However, the explored area of α5 was 2.1 and 3.4 fold larger than that of γ2 in extrasynaptic region and at inhibitory synapses, respectively (KS test, p<10^−4^ and p = 10^−3^, respectively; Fig. 1D and Table 1), indicating a stronger confinement for γ2.

**Table 1:** Diffusion properties of GABAAR γ2 and α5 subunits, and of AMPAR GluA2 subunit in relation with excitatory and inhibitory synapses.

	Location	Median D (10^−2^ µm^2^s^−1^)	Median EA (10^−3^ µm^2^)	Dwell time (s)
	In. Sy.	0.7 (687)	8.7 (132)	7.1±0.8 (251)
**GABA_A_R γ2**	Exc. Sy.	1.2 (510)	21.6 (147)	2.5±0.3 (138)
	Non Sy.	1.5 (575)	26.2 (1992)	*n.d.*
	In. Sy.	1.4 (124)	28.6 (57)	2.4±0.8 (78)
**GABA_A_R α5**	Exc. Sy.	1.8 (68)	6.8 (81)	1.7±0.2 (116)
	Non Sy.	2.8 (436)	54.9 (1371)	*n.d.*
	In. Sy.	1.9 (103)	32.42 (201)	2.1±0.3 (177)
**GluA2**	Exc. Sy.	1.9 (228)	14.45 (241)	3.6±0.5 (321)
	Non Sy.	5.6 (786)	116.4 (3036)	*n.d.*

Median D: median diffusion coefficient, Median EA: median explored area. In. Sy.: Inhibitory Synapses, Exc. Sy.: Excitatory Synapses, Non Sy.: Non Synaptic, n.d.: not determined. Values of dwell time are mean ± SEM. Quantifications were from 10 cultures for GABAAR γ2, 6 cultures for α5 and 3 cultures for GluA2 (n between parentheses).

**Figure 2:**
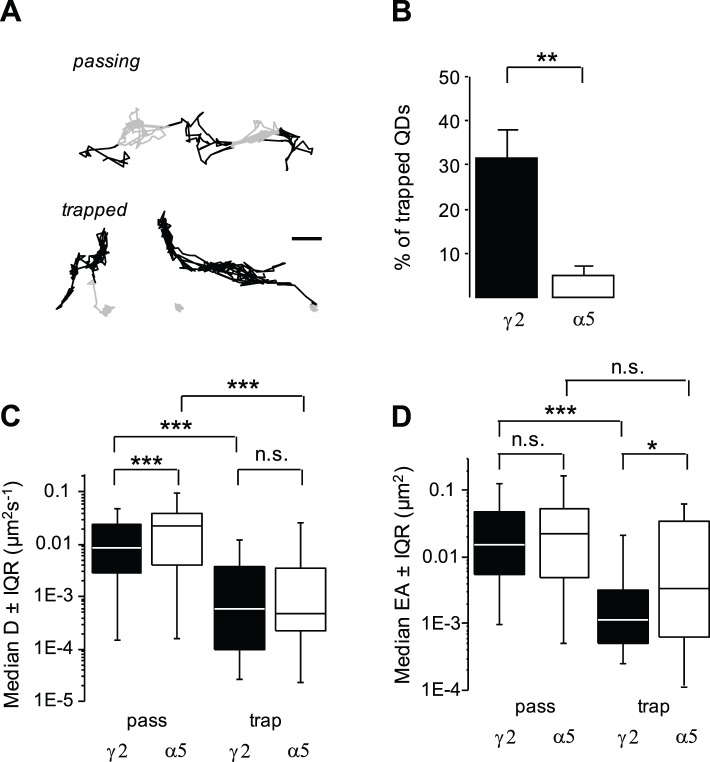
Segregation of passing and trapped GABA_A_R γ2 and α5 at inhibitory synapses. (A) Examples of GABA_A_R γ2 trajectories at inhibitory synapses for passing (top) and trapped (bottom) receptors outside (black) or inside (grey) synapses. Scale, 0.5 µm. (B) Proportion (mean ± SEM) of γ2 (black) and α5 (white) trajectories classified as trapped at inhibitory synapses (t-test, **p<10^−2^). (C) Trapped (trap) γ2 (black) and α5 (white) have lower mobility (median D ±25%–75% IQR) in inhibitory synapses than passing ones (KS test, ***p<10^−3^). (D) Passing (pass) γ2 (black) and α5 (white) explored a similar surface area (median EA ±25%–75% IQR) while trapped γ2 explored a smaller surface area than α5 (KS test, *p<5×10^−2^; ***p<10^−3^).

Since the time receptors spend at the synapse influences synaptic receptor content [2], we compared the synaptic dwell time of GABA_A_R γ2 and α5 subunits at inhibitory synapses. The mean dwell time of γ2 was 3-fold that of α5 (t-test, p<10^−4^; Fig. 1F), indicating a faster exchange of α5 between extrasynaptic and synaptic locations. This is consistent with the notion that γ2 but not α5 is enriched at inhibitory synapses. Actually, this means that the dwell time of α5 reflected the time required to get across an inhibitory synapse without being trapped by the scaffolding apparatus. As 95% of α5 trajectories had dwell time values less than 5.9 s, we used this threshold to segregate “trapped” receptors (dwell time>5.9 s) from “passing” ones (dwell time< = 5.9 s) (e.g. Fig. 2A). In agreement with a higher accumulation of γ2 at inhibitory synapses, a larger proportion of γ2 than α5 was trapped at inhibitory synapses (γ2, 31.7±6.4%; α5, 5.1±2.0%; t-test, p = 0.04; Fig. 2B). Surprisingly, the proportion of trapped γ2 was in minority at inhibitory synapses meaning that most γ2 were passing or were interacting only transiently with the scaffold.

The median diffusion of γ2 and α5 was the same for trapped receptors (γ2, median D = 0.06×10^−2^ ****µm^2^s^−1^, n = 84; α5, median D = 0.05×10^−2^ ****µm^2^s^−1^, n = 8; KS test, p = 0.67; Fig. 2C). In contrast, the diffusion of passing receptors containing the γ2 subunit was significantly reduced (γ2, median D = 0.87×10^−2^ ****µm^2^s^−1^, n = 255; α5, median D = 2.32×10^−2^ ****µm^2^s^−1^, n = 56; KS test, p<0.01; Fig. 2C). However, this did not have a significant impact on the synaptic area explored by passing receptors (γ2, median area = 15.1×10^−3^ ****µm^2^; n = 244; α5, area = 22.4×10^−3^ ****µm^2^; n = 39; KS test, p = 0.54; Fig. 2D). Interestingly, trapped receptors containing γ2 explored a smaller area of the synaptic compartment than α5 (γ2, area = 1.2×10^−3^ ****µm^2^; n = 79; α5, area = 4.4×10^−3^ ****µm^2^; n = 12; KS test, p<0.05; Fig. 2D), suggesting increased constraints for γ2 such as scaffold interaction.

Altogether our results suggest the reduction of the mobility of α5 at inhibitory synapses involve obstacles and fences while that of γ2 further implicate receptor-scaffold interactions. Furthermore, the dwell time but not the diffusion coefficient calculated on the global population of synaptic QDs (independent of their trapped vs passing behavior) allows distinguishing GABA_A_Rs accumulated (γ2) or not (α5) at synapses.

### Diffusion of the GABA_A_Rs γ2 and α5 Subunits at Excitatory Synapses

**Figure 3:**
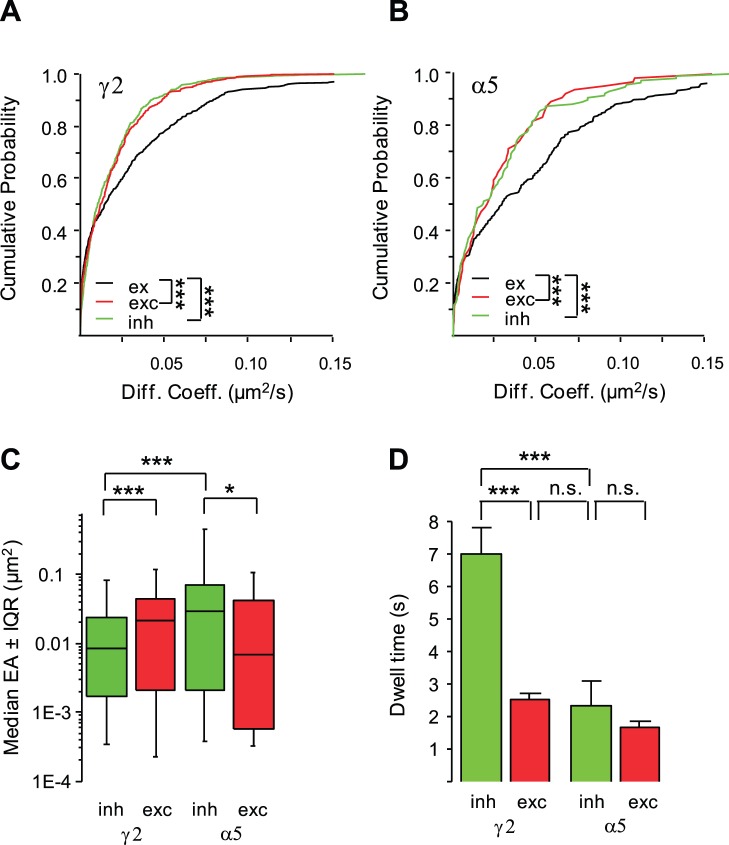
Comparison of the diffusion properties of GABA_A_R γ2 and α5 in inhibitory and excitatory synapses. (A–B) Cumulative probabilities of diffusion coefficients of γ2 and α5 in inhibitory (green, inh) and excitatory synapses (red, exc) *vs*. the extrasynaptic membrane (black, ex). Note the reduced diffusion of γ2 and α5 in inhibitory and excitatory synapses as compared to the extrasynaptic membrane (KS test, ***p<10^−3^). (C–D) Decreased explored surface area (C, median EA ±25%–75% IQR; KS test, *p<5×10^−2^; ***p<10^−3^) and dwell time (D, mean ± SEM; t-test, ns: not significant, ***p<10^−3^) for γ2 in inhibitory (green) *vs*. excitatory (red) synapses. In contrast, the dwell time of α5 did not significantly differ in inhibitory (green) and in excitatory (red) synapses.

We then asked whether the membrane diffusion of GABA_A_R γ2 and α5 subunits was reduced within excitatory postsynaptic differentiations. The localization of QD trajectories at excitatory synapses was determined by comparison with merge fluorescence images of venus-gephyrin and FM 4–64. QD trajectories were respectively at inhibitory or at excitatory synapses when overlapping with FM 4–64 loaded presynaptic boutons colocalized or not with recombinant venus-gephyrin clusters. Trajectories localized on areas devoid of FM 4–64 and venus-gephyrin labeling were considered extrasynaptic. Relative to extrasynaptic regions, the lateral diffusion of γ2 and α5 was reduced in excitatory synapses (10 cultures; KS test, p<10^−4^; α5, 6 cultures; KS test, p<10^−3^; Fig. 3 A–B; Table 1). Furthermore, the diffusion coefficient of γ2 and α5 did not significantly differ in excitatory and inhibitory synapses (KS test, p = 0.2 and 0.8 respectively; Fig. 3 A–B; Table 1). However, the reduction of the explored area of γ2 was more pronounced in inhibitory *vs*. excitatory synapses (KS test, p = 0.0002; Fig. 3C; Table 1). Furthermore, γ2 resided ∼2.9-fold longer in inhibitory synapses (t-test, p<10^−3^; Fig. 3D; Table 1), indicating a faster escape from excitatory synapses. This is coherent with the notion that γ2 is anchored to the inhibitory but not to the excitatory postsynaptic scaffold. In contrast, the dwell time of α5 was not significantly different at excitatory and at inhibitory synapses (t-test, p = 0.3; Fig. 3D; Table 1). Interestingly, the average synaptic dwell time of α5 at inhibitory synapses was close to the one found for γ2 at excitatory synapses (t-test, p = 0.8; Fig. 3D; Table 1). We therefore concluded that the diffusion of γ2 and α 5 was also hindered by the presence of obstacles in excitatory synapses. Moreover, the dwell time but not the diffusion coefficient calculated on the entire population of synaptic QDs unraveled the sorting of GABA_A_Rs at inhibitory synapses.

**Figure 4:**
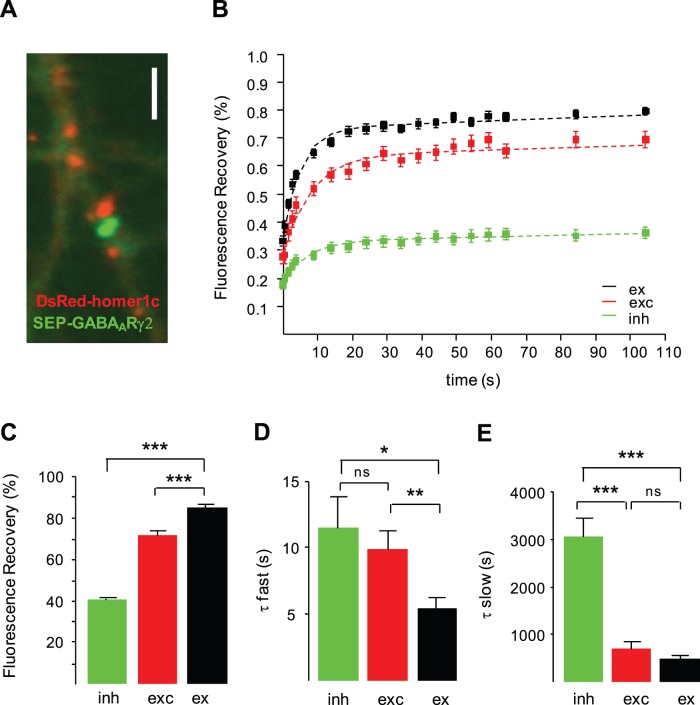
Fluorescence recovery after photobleaching experiments of SEP-GABA_A_R γ2 at excitatory and inhibitory synapses. (A) Dendritic portion of a neuron co-transfected with SEP-GABA_A_Rγ2 (green) and DsRed-homer1c (red) constructs. Scale, 1 µm. Note that SEP-GABA_A_R γ2 (green) form clusters that are not colocalized with DsRed-homer1c (red) fluorescent clusters. (B) Normalized SEP-GABA_A_Rγ2 FRAP fluorescence recovery curves at inhibitory synapses (green, inh), excitatory synapses (red, exc), and in extrasynaptic compartment (black, ex). (C) Percentage (mean ± SEM) of SEP-γ2 fluorescence recovery at inhibitory synapses (inh, green), excitatory synapses (exc, red) and extrasynaptically (ex, black). Note the increase in the size of the pool of slowly mobile γ2 at excitatory synapses as compared with the extrasynaptic compartment (t-test, ***p<10^−3^). (D–E) Time constants (mean ± SEM) for the fast (D) and slow (E) pool, obtained from the double exponential fit (see Materials and Methods) applied to the data in B for inhibitory (green, inh), excitatory (red, exc) synapses or extrasynaptic area (black, ex) (t test, ns: not significant, *: p<0.05, **: p<0.01; ***: p<0.001).

To verify that the constrained diffusion of GABA_A_R γ2 at excitatory synapses was not due to the presence of QDs, we performed Fluorescence Recovery After Photobleaching (FRAP) experiments of the sensitive superecliptic pHluorin GABA_A_R γ2 subunit (SEP-γ2) in neurons double-transfected with Red fluorescent protein from *Discosoma* species (DsRed)-tagged homer1c. SEP-γ2 and DsRed-homer1c fluorescent punctae (Fig. 4A) identified inhibitory and excitatory synapses, respectively. SEP-γ2 fluorescence was ∼3-fold higher in inhibitory *vs*. excitatory synapses or in extrasynaptic membrane (Fig. S1), indicating a specific concentration at inhibitory synapses. The SEP fluorescence is pH-sensitive: it exhibits bright fluorescence when exposed at the cell surface and lower fluorescence in internal acidified trafficking organelles [33]. As shown before [16], the SEP-γ2 fluorescence specifically reported surface γ2 since neuronal exposure to pH4 buffer caused an instantaneous loss of nearly all fluorescence without main changes in monomeric Red Fluorescent Protein (mRFP)-tagged gephyrin fluorescence in co-transfected neurons (Fig. S1). Moreover, the eclipsed fluorescence rapidly returned in pH 7.4 buffer (Fig. S1). After photobleaching, the fluorescence of SEP-γ2 clusters recovered to ∼35% (34.3±1.3, n = 65) of the initial value within 2 min (Fig. 4 B), meaning that most SEP-γ2 at inhibitory synapses was part of a stable pool that did not exchange during the course of the experiment. This contrasted with the fluorescence recovery in the extrasynaptic membrane which reached 83±1% of the initial fluorescence value within 1 min (Fig. 4B). In other words, the stable pool of SEP-γ2 represented most (60%) of the postsynaptic receptors whereas it corresponds to a minority (15%) of the whole extrasynaptic population. Interestingly, the stable pool of SEP-γ2 at excitatory synapses was twice as much the stable pool of receptors in the extrasynaptic membrane. This difference was highly significant (t-test, p<10^−3^; Fig. 4C). In line with the SPT data, these results indicate higher diffusion constraints on γ2 at excitatory synapses as compared with the extrasynaptic membrane. Therefore, molecular constraints rather than QD detection method account for the increased confinement of γ2 at excitatory synapses.

FRAP curves were then fitted with a double exponential function with two different kinetic constants (fast and slow), as previously done [34], [35]. We found that the fast pool recovered faster at extrasynaptic location as compared with excitatory and inhibitory synapses (extrasynaptic: 5.44±0.81 s; inhibitory synapses: 11.46±2.40 s; t test p = 0.04, excitatory synapses: 9.87±1.36 s, t test p = 0.008; Fig. 4D), suggesting increased diffusion constraints for SEP-γ2 at both excitatory and inhibitory synapses. The significant reduction in the fast pool recovery at excitatory synapses vs the extrasynaptic compartment suggests the fast pool reports on freely diffusing receptors and receptors encountering weak molecular constraints. Interestingly, the time constant of the fast pool was not significantly different at excitatory vs inhibitory synapses (p = 0.43; Fig. 4D). In contrast, for the slow pool the slowest recovery was obtained at inhibitory synapses (extrasynaptic: 467±90 s; inhibitory synapses: 3059±396 s p = 0.0003; excitatory synapses: 695±141 s, p = 0.17; Fig. 4E). The time constant of the slow pool did not significantly differ at excitatory synapses and at extrasynaptic site (Fig. 4E), meaning that, contrary to inhibitory synapses, γ2 did not encounter strong constraints at excitatory ones. Therefore, FRAP results confirmed our SPT data demonstrating that γ2 encountered weak constraints at both excitatory and inhibitory synapses whereas the strong constraints appeared only at inhibitory synapses.

### Impact of Overexpression of Gephyrin Dominant-negative Constructs on the Lateral Diffusion of the GABA_A_R γ2 Subunit

**Figure 5:**
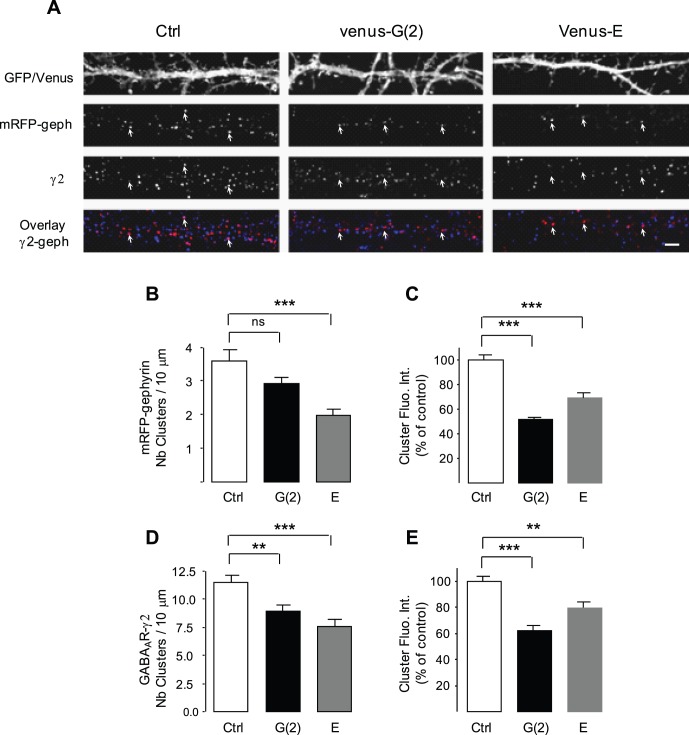
Gephyrin dominant-negative chimera decreased gephyrin and GABA_A_R γ2 clustering. (A) Hippocampal cultured neurons from mRFP-gephyrin knock-in mice transfected with GFP, venus-G(2), or venus-E constructs, and stained for γ2. Scale, 2 µm. Note that mRFP-gephyrin and GABA_A_R γ2 formed numerous clusters along dendrites of GFP transfected neurons. Many mRFP-gephyrin clusters were colocalized with GABA_A_R γ2 clusters (arrows). Over-expression of the venus-G(2) and venus-E chimera resulted in a marked reduction of the number and intensity of mRFP-gephyrin and GABA_A_R γ2 clusters. The remaining mRFP-gephyrin clusters were rarely colocalized with γ2 clusters (arrows). (B-E) Quantifications showing venus-G(2) and venus-E constructs reduced both the number of gephyrin (B) and γ2 (D) clusters per 10 µm dendritic length and the fluorescence intensity of the corresponding clusters (C, E). Values are mean ± SEM. t-test, ns: not significant, **: p<0.01; ***: p<10^−3^.

We then studied the role of the main inhibitory scaffolding molecule gephyrin in the lateral diffusion of γ2 at inhibitory synapses. The gephyrin sequence contains amino and carboxy terminal G and E domains, connected by a central linker C [36]. Expression of the isolated G and E domains of gephyrin interfere with the oligomerization and synaptic clustering of the full length scaffolding protein in spinal cord neurons [37]. We first analyzed the impact of venus-tagged G(2) and E domains on gephyrin clustering in hippocampal neurons dissociated from mRFP-gephyrin knock-in mouse allowing direct imaging of mRFP-gephyrin clusters [38]. Neurons were transfected with venus-G(2), venus-E chimera or with a plasmid encoding Green Fluorescent Protein (GFP) alone for a control. In control conditions, mRFP-gephyrin formed numerous clusters along dendrites of GFP transfected neurons (Fig. 5A), and many of them were colocalized with GABA_A_R γ2 clusters (overlay, Fig. 5A). As reported previously [37], the isolated G(2) and E domains were diffusely distributed in the cytoplasm of transfected neurons (Fig. 5A). Overexpression of the G(2) and E domains noticeably reduced the number of mRFP-gephyrin and GABA_A_R γ2 clusters as well as the fluorescence intensity of the remaining clusters (Fig. 5A). As seen on the overlays, gephyrin clusters were rarely colocalized with GABA_A_R γ2 staining in neurons transfected with the dominant-negative constructs (Fig. 5A). Isolated G(2) domains decreased the intensity of the remaining gephyrin clusters by ∼2 fold, (73–78 cells, 2 cultures; t test, p<0.0001; Fig. 5B–C). Overexpression of the E domain also efficiently interfered with gephyrin aggregation, decreasing the number per 10 µm dendritic length of gephyrin clusters and their intensity (73–82 cells, 2 cultures; t test, p = 0.0002 and p<0.0001; Fig. 5B–C). However, the influence of the G(2) and E domains were more pronounced respectively on the fluorescence intensity of gephyrin clusters and the density of gephyrin clusters. These data demonstrate that the isolated G(2) and E domains exert a dominant-negative effect on the clustering of mRFP-gephyrin in hippocampal neurons. This is reminiscent of what was found in spinal cord neurons [37]. In parallel, the isolated G(2) and E domains efficiently reduced the density of γ2 clusters (t test, p = 0.001 and p<0.0001, respectively; Fig. 5D) and the fluorescence intensity of γ2 clusters (t test, p<0,0001 and p = 0.0027, respectively; Fig. 5E), consistent with the notion that γ2 containing GABA_A_Rs require gephyrin for their clustering [16], [39], [40], [41], [42].

**Figure 6:**
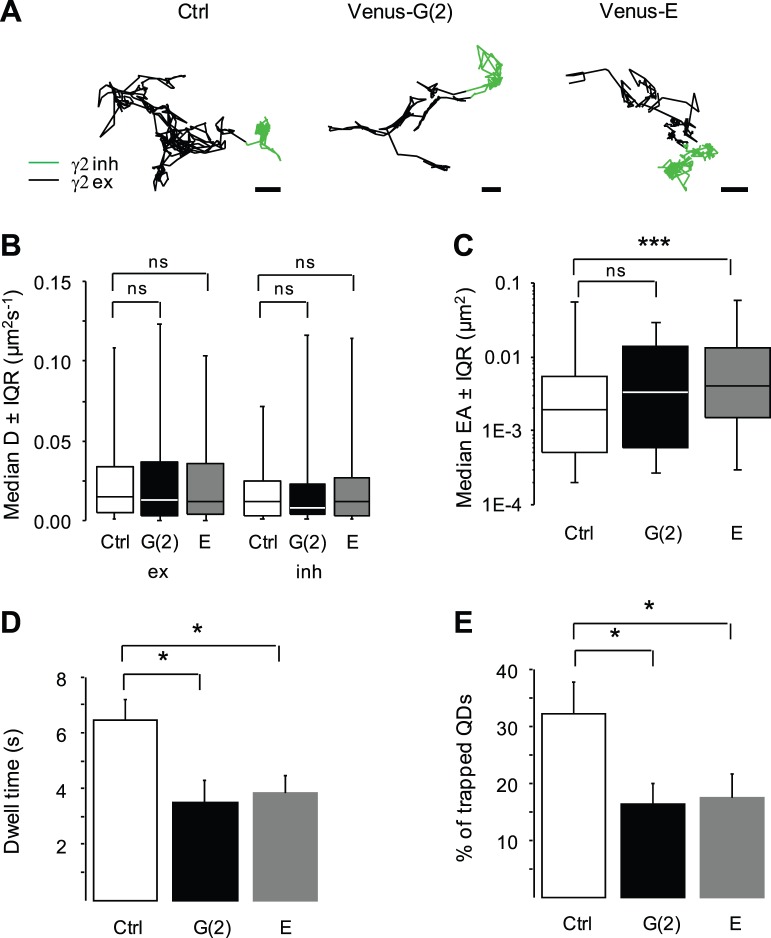
Effects of gephyrin clustering interference on the diffusion properties of the GABA_A_R γ2 subunit at inhibitory synapses. (A) Individual trajectories of QDs coupled to γ2 outside (black, ex) or inside (green, inh) inhibitory synapses in mRFP-gephyrin knock-in mice neurons transfected with GFP, venus-G(2) or venus-E constructs. Scale, 0.5 µm. (B) Over-expression of the venus-G(2) (black) or venus-E (grey) chimera did not change the diffusion coefficient (median D ±25%–75% IQR; KS test, ns: not significant) of γ2 outside (ex) or inside (inh) inhibitory synapses as compared to the respective control conditions (GFP, white). In contrast, the gephyrin dominant-negative venus-G(2) (black) and venus-E (grey) constructs increased the explored surface area (C, median EA ±25%–75% IQR; KS test, ns: not significant, ***: p<10^−3^), and decreased the dwell time (D, mean ± SEM; t test *: p<0.05) and the proportion of trapped γ2 (E, mean ± SEM; t test *: p<0.05) at inhibitory synapses.

We next investigated the effects of the gephyrin dominant-negative constructs on the lateral diffusion of GABA_A_R γ2. Trajectories were considered as synaptic when overlapping with mRFP-gephyrin clusters (e.g. green trajectory in Fig. 6A). Overexpression of the isolated G(2) and E domains did not change the diffusion coefficient of the entire population (trapped + passing) γ2at inhibitory synapses 2 cultures; KS test p = 0.11 and p = 0.34; Fig. 6B and Table 2) or in extrasynaptic membranes 2 cultures; KS test p = 0.06 and p = 0.06; Fig. 6B and Table 2). In contrast, the chimeras increased the γ2 explored area at inhibitory synapses (Fig. 6C and Table 2), showing reduced confinement. This effect was more pronounced with the E domain (t test p<0.0001 and p = 0.2 for the E and G(2) domains, respectively). Moreover, the expression of both chimera reduced by ∼2 fold the dwell time (t test, p<0.05; Fig. 6D and Table 2) and the proportion of trapped γ2 (t test, p<0.05; Fig. 6E and Table 2) at inhibitory synapses. Thus, the explored area and dwell time but not diffusion coefficient of the whole population of QDs are correlated with gephyrin-mediated trapping and accumulation of γ2 at inhibitory synapses.

**Table 2:** Effects of gephyrin dominant negative chimera on the diffusion properties of the GABAAR γ2 subunit.

Transfection	Location	Median D (10^−2^ µm^2^s^−1^)	Median EA (10^−3^ µm^2^)	Dwell time (s)
**Ctrl**	Non Sy	1.47 (821)	25.54 (5229)	*n.d.*
**G(2)**	Non Sy.	1.30 (418)	15.4 (1083)	*n.d.*
**E**	Non Sy.	1.12 (904)	16.41 (3675)	*n.d.*
**Ctrl**	In. Sy.	1.21 (103)	1.97 (194)	6.4±0.8 (221)
**G(2)**	In. Sy.	0.82 (96)	3.33 (82)	3.5±0.8 (58)
**E**	In. Sy.	1.18 (169)	4.10 (202)	3.8±0.6 (94)

Median D: median diffusion coefficient, Median EA: median explored area. In. Sy.: Inhibitory Synapses, Exc. Sy.: Excitatory Synapses, Non Sy.: Non Synaptic, n.d.: not determined. Values of dwell time are mean ± SEM. Quantifications were from 2 independent cultures (n between parentheses).

### Diffusion of the GluA2 Subunit of the AMPA Receptor at Excitatory and Inhibitory Synapses

**Figure 7:**
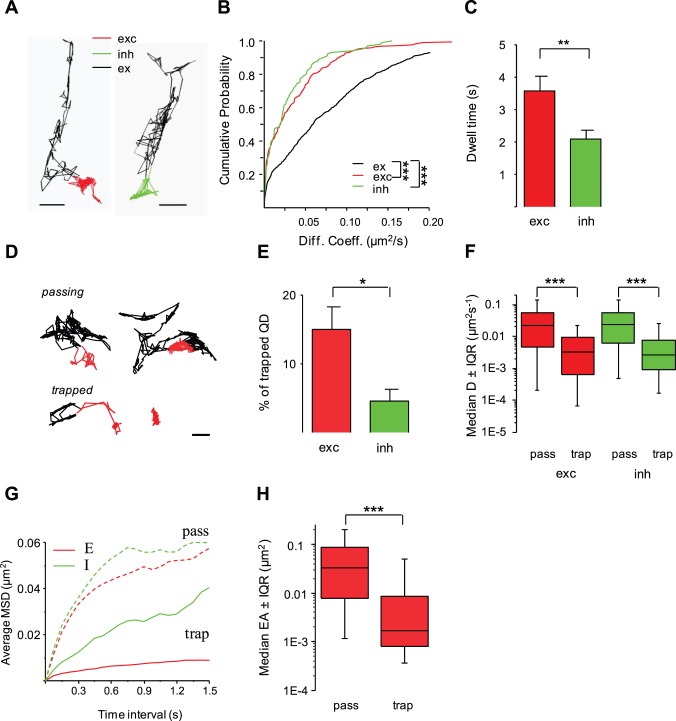
Diffusion characteristics of the GluA2 subunit of the AMPAR in inhibitory and excitatory synapses. (A) Trajectories of GluA2-coupled QDs in excitatory (red), inhibitory (green) synapses or in extrasynaptic compartment (black). Scale, 0.4 µm. (B) Cumulative probabilities of diffusion coefficients of GluA2 in inhibitory (green) and excitatory synapses (red) *vs*. the extrasynaptic membrane (black) (KS test, ***p<10^−3^). (C) Dwell time (mean ± SEM) of GluA2 in excitatory (exc, red) or inhibitory (inh, green) synapses (t test **p<10**^−^** ^2^). (D) Representative examples of trajectories for passing (top) and trapped (bottom) receptors outside (black) or inside (red) excitatory synapses. Scale, 0.3 µm. (E) Increased proportion (mean ± SEM) of trapped GluA2-coupled QDs in excitatory (exc, red) *vs*. inhibitory (inh, green) synapses (t test, *p<5×10**^−^** ^2^). (F) Reduced mobility (median D ±25%-75% IQR; KS test, ***p<10^−3^) for trapped (trap) receptors in excitatory (exc, red) and inhibitory (inh, green) synapses *vs*. passing ones (pass). (G) Averaged MSD *vs*. time relation for passing (broken line) or trapped (plain line) GluA2 in excitatory (red) or inhibitory (green) synapses. (H) Explored area (median EA ±25%–75% IQR) for trapped (trap) receptors at excitatory synapses (exc, red) (KS test, ***p<10^−3^).

We reasoned that excitatory receptors should also be restricted in their diffusion at mismatched synapses. QD-based SPT experiments of the GluA2 subunit of the α-amino-3-hydroxy-5-methyl-4-isoxazolepropionic acid receptor (AMPAR) were performed in neurons transfected with GFP-coupled homer1c and mRFP-gephyrin to label excitatory (GFP-homer1c fluorescent clusters) and inhibitory (mRFP-gephyrin fluorescent punctae) synapses, respectively. As visualized on individual trajectories (e.g. Fig. 7A), the surface exploration of QDs decreased at excitatory and inhibitory synapses as compared with the extrasynaptic membrane. As shown for these examples, the diffusion dropped while the confinement increased when QDs went across excitatory and inhibitory synapses (Fig. S2). Quantitative analysis performed on a large number of QDs confirmed GluA2 was significantly slowed down when passing through synapses whatever the neurotransmitter contained in the presynaptic element (3 cultures; KS test, excitatory synapses *vs*. extrasynaptic, p<10^−3^; inhibitory synapses *vs*. extrasynaptic, p<5×10^−2^; Fig. 7B; Table 1). Furthermore, GluA2 diffusion was not significantly different at excitatory *vs*. inhibitory synapses when considering the whole QDs population (KS test, p = 0.5). In contrast, the dwell time of GluA2 was increased by ∼1.2-fold at excitatory synapses (t-test, p<10^−2^; Fig. 7C; Table 1). Using the criteria of distinction of trapped *vs*. passing QDs, we found the exploratory behavior of trapped GluA2 was restricted to a smaller area of the excitatory synapse (Fig. 7 D, G–H), indicating increased confinement. The proportion of GluA2 trapped at excitatory synapses (15±3.3%, n = 52) was 3.3-fold that found at inhibitory synapses (4.6±1.7%, n = 37; Fig. 7E), informing on local anchoring. It is worth noting that most GluA2 containing AMPARs encountered weak molecular constraints at excitatory synapses since only 15% of the QDs were trapped (Fig. 7E). Relative to passing GluA2, the diffusion of trapped GluA2 was reduced at excitatory synapses (trapped, median D = 0.4×10^−2^ ****µm^2^s^−1^, n = 71; passing, median D = 2.3×10^−2^ ****µm^2^s^−1^, n = 303; 3 cultures; KS test, p<10^−4^; Fig. 7F) and at inhibitory synapses (trapped, median D = 0.3×10^−2^ ****µm^2^s^−1^, n = 39; passing, median D = 2.3×10^−2^ ****µm^2^s^−1^, n = 146; 3 cultures; KS test, p<10^−4^; Fig. 7F). As expected, trapped GluA2 explored smaller areas (trapped, median area = 1.9×10^−3^ ****µm^2^ n = 90; passing, median area = 32×10^−3^ ****µm^2^; n = 151; 3 cultures; KS test, p<10^−4^; Fig. 7G–H).

Altogether, our data show GABA_A_R and AMPAR are significantly hindered in their diffusion at mismatched synapses, and the confinement and dwell time but not the diffusion coefficient report on local anchoring at matched synapses.

## Discussion

The main conclusions of this work are that i) diffusing neurotransmitter receptors do cross PSDs where they do not usually accumulate, ii) obstacles and fences significantly reduce the lateral diffusion of neurotransmitter receptors at synapses, and iii) the explored area and dwell time but not the diffusion coefficient inform on synapse specific sorting, trapping and accumulation of receptors.

### Relevant Diffusion Parameters for Synaptic Sorting, Trapping and Accumulation of Receptors

We found that the measurement of diffusion coefficients on the whole population of QDs (trapped + passing) did not allow distinguishing receptors slowdown by diffusion barriers and obstacles from receptors interacting with the scaffold. This was shown for GABA_A_R γ2 and AMPAR GluA2 subunits by comparing the cumulative distributions of diffusion coefficients at excitatory vs inhibitory synapses. Furthermore, we showed that preventing GABA_A_R γ2 and gephyrin interaction with a dominant negative approach did not change the diffusion coefficients of γ2. This observation was unexpected since receptor-scaffold interactions do lead to a significant reduction in the diffusion coefficient of GABA_A_R (Fig. 2) and AMPARs (Fig. 7) trapped at the synapse as compared to passing ones. This is in agreement with previous data showing a shift in the diffusion coefficient toward lower values for receptors diffusing in the scaffolding molecule enriched zone (e.g. GlyR-gephyrin: [43]; GABA_A_R-gephyrin: [44]; mGluR-homer: [45]; GluA1 AMPAR-PSD95: [46]). However, we compared the distribution of diffusion coefficients on the whole population of QDs detected at synapses, independently of their trapped vs passing behavior. We showed here that most receptors detected at matched synapses are passing receptors i.e. receptors not captured by the scaffold or receptors interacting only transiently with the scaffold. Indeed, we found that only ∼30% of GABA_A_R γ2 and ∼15% of GluA2 were trapped at inhibitory synapses and excitatory synapses respectively. The predominance of passing receptors at synapses thus condition the average behavior of QDs. Moreover, the changes in mobility resulting from the transitions between states such as bound and unbound to a scaffolding element can be difficult to identify with the classical MSD analysis (refs in [47]). Therefore, when looking at global population of QDs, the diffusion coefficient measurement does not inform on receptor anchoring. Calculation of diffusion coefficient over a sliding window (Dinst, Figs. 1C and F) could be more appropriate, but the error in the calculation of the diffusion coefficient is very important for short trajectories [48]. Therefore, Dinst does not provide a more realistic value because of its high uncertainty.

Interestingly, GluA2 was more mobile, explored a larger area and resided shorter time in excitatory synapses relative to GABA_A_Rs at inhibitory synapses. AMPARs are more mobile within excitatory synapses than NMDA receptors [49], [50]. GABA_A_Rs also display lower diffusion constraints than glycine receptors (GlyR) in inhibitory synapses formed between spinal cord neurons [18]. These differences are likely attributable to differences in binding affinity to the postsynaptic scaffold. Actually there was a higher proportion of trapped GABA_A_Rs (∼30%) than AMPARs (15%), suggesting that the affinity of AMPARs for their scaffolding molecules is lower than that of GABA_A_Rs.

Furthermore, we found that the median explored area values were very variable in extrasynaptic membranes for GABA_A_R γ2, GABA_A_R α5 and GluA2 (Table 1). These differences were accompanied by variations in the median diffusion coefficient values. These differences in mobility indicate that not all receptors are equivalent with respect to diffusion in extrasynaptic membrane i.e. for their capacity to interact with obstacles and diffusion barriers. This can be due to various incorporations into multimolecular complexes that impact the size of the tracked molecules, or various interactions with extrasynaptic scaffolding molecules.

### Barriers to Diffusion and Receptor Accumulation at Synapses

Reduced diffusion of lipids [35] and neurotransmitter receptors (the present study) at both excitatory and inhibitory synapses confirm the presence of diffusion barriers at both types of synapses. Although constraining the diffusion of receptors, the diffusion barriers at synapses are permeable. Our report of constrained diffusion of GABA_A_R γ2 and α5 at excitatory synapses and AMPAR at inhibitory synapses is reminiscent of what was found for the lipid rafts markers, cholera toxin (ChTx) and glycophosphatidylinositol-anchored green fluorescent protein (GPI-GFP) and the non raft marker, Phosphatidylethanolamine (DOPE) [14], [35].

Lipids are associated with the outer part of the membrane leaflet while receptors have intracellular tails that protrude below the membrane. Based on the analysis of the mobility of lipids at synapses, it was proposed that the diffusion is more constrained at inhibitory synapses than at excitatory ones. Indeed, the diffusion of lipids dropped, and their confinement and dwell time increased at inhibitory synapses *vs*. excitatory ones [35]. On the contrary, we found the GABA_A_R α5 subunit, not enriched at synapses, had similar diffusion coefficient and dwell time at both inhibitory and excitatory synapses. This means the permeability of the barrier varies in function of the nature of the diffusing molecule (membrane anchored *vs*. membrane spanning). A high density in different molecular components of the barrier may define its permeability and its function. For instance, an increased density of specific isoforms of ankyrin G and spectrin are required for the segregation of the axonal and the dendritic plasma membrane at the axon initial segment [51]. Furthermore, the scaffolding molecule septin7, enriched at the base of dendritic spines may act as a diffusion barrier to physically separate and compartmentalize dendritic shaft and spines [52].

The reduction in diffusion does not lead to the protein accumulation at synapses if the net flux of molecules equals zero (i.e. at steady state, see [2]). However, obstacles may occasionally permit the local accumulation of proteins by increasing their confinement and dwell time. Receptors have been occasionally found clustered at mismatched synapses. In the cerebellum, the GABA_A_R α6, γ2, and β2/3 subunits are concentrated at the inhibitory Golgi synapses as well as at some excitatory glutamatergic mossy synapses together with functional AMPA-type glutamate receptors [53], [54]. It was proposed that GABA_A_R clustering at excitatory postsynaptic locus mediates inhibition via GABA spillover from nearby Golgi terminals [53], [55]. Therefore obstacles may have a functional implication in synaptic transmission. This will be a way for some synapses to use both GABAergic and glutamatergic signaling without neurotransmitter co-release from the same terminal.

## Materials and Methods

### Ethics Statement

All animal procedures in this study were performed in accordance with the guidelines issued by the French Ministry of Agriculture and approved by the Direction Départamentale des services Vétérinaires de Paris (Ecole Normale Supérieure, Animalerie des Rongeurs, license B 75-05-20). All efforts were made to minimize animal suffering and to reduce the number of animals used.

### Cell Culture and Transfection

Primary cultures of hippocampal neurons were prepared as described [56] with some modifications of the protocol. Briefly, hippocampi were dissected from embryonic day 18 or 19 Sprague-Dawley rats or from embryonic day 17 mRFP-gephyrin knock-in mice for experiments involving the gephyrin dominant-negative approach (Fig. 5 and 6). Tissue was then trypsinized (0.25% v/v), and mechanically dissociated in 1× HBSS (Invitrogen, Cergy Pontoise, France) containing 10 mM HEPES (Invitrogen). Neurons were plated at a density of 2.3 ×10^4^ cells/cm^2^ (rat cultures) or 3.9 ×10^4^ cells/cm^2^ (mRFP-gephyrin knock-in mice cultures) onto 18-mm diameter glass coverslips (Assistent, Winigor, Germany) precoated with 80 µg/ml poly-D,L-ornithine (Sigma, St Louis, MO) in plating medium composed of minimum essential medium (MEM, Sigma) supplemented with horse serum (10% v/v, Invitrogen), L-glutamine (2 mM) and Na+ pyruvate (1 mM) (Invitrogen). After attachment for 2–3 hours, cells were incubated in maintenance medium that consists of Neurobasal medium supplemented with B27 (1X), L-glutamine (2 mM), and antibiotics (Invitrogen) for up to 3 weeks at 37°C in a 5% CO2 humidified incubator. At day 7 in vitro (DIV), one fifth of maintenance medium supplemented with horse serum (5% v/v) was added. At DIV 14 and 21, one sixth of maintenance medium was renewed. Transfections with venus-gephyrin, mRFP-gephyrin [32], GFP-homer1c, DsRed-homer1c [46], SEP-GABA_A_Rγ2 [16], venus-G(2), venus-E [37] or GFP (pEGFP, Clontech) chimeras were done at DIV 13–15 using the Lipofectamine 2000 method (Invitrogen) according to the manufacturer’s instructions, with 0.5–1 µg of plasmid DNA per 20 mm wells. Experiments were performed two days after transfection. A plasmid equimolar ratio of cDNA was used in all cotransfection experiments.

### Immunocytochemistry

Cells were fixed for 15 min with paraformaldehyde (4% w/v, Serva Feinbiochemica, Heidelberg, Germany) in PBS containing sucrose (4% w/v), washed, quenched with NH_4_Cl (33 mM) in PBS and permeabilized for 4 min with Triton X-100 (0.25% v/v) in PBS. After washes, nonspecific staining was blocked for 30 min with gelatin (0.25% w/v, Sigma) in PBS. Cultures were incubated for 1 hr with primary antibodies in PBS supplemented with gelatin (0.125% w/v), washed and incubated for 45 min with secondary antibodies. After washes, coverslips were mounted onto glass slides using Vectashield (Vector Laboratories, Burlingame, CA). All washes and incubation steps were performed at room temperature in PBS supplemented with gelatin. The primary antibodies used were guinea-pig anti-GABA_A_R α5 subunit (1∶1000; gift from J.M. Fritschy, Unniversity of Zurich, Zurich, Switzerland), guinea-pig anti-GABA_A_R γ2 subunit (1∶2000; gift from J.M. Fritschy), rabbit anti- GABA_A_R γ2 subunit (1∶100; Alomone Labs, Jerusalem, Israel), mouse anti-gephyrin (mAb7a, 1.25 µg/ml; Synaptic Systems, Gottingen, Germany) and mouse anti-GluA2 (1∶200; BD Pharmingen, Franklin Lakes, USA). Secondary antibodies were Cy3 conjugated goat anti- mouse, rabbit, or guinea pig (3.75 µg/ml), FITC-conjugated goat anti-mouse (3.75 µg/ml) from Jackson ImmunoResearch (West Grove, PA). For GABA_A_R γ2 and α5 subunits immunodetection, live neurons were washed at 37°C with imaging medium composed of phenol-red free minimum essential medium (MEM, Invitrogen) supplemented with glucose (33 mM; Sigma), HEPES buffer (20 mM), L-glutamine (2 mM), Na^+^ pyruvate (1 mM), and B27 supplement (1X) (Invitrogen), and incubated for 30 min at 37°C with primary antibodies in imaging medium. Following washes with imaging medium, cells were fixed for 15 min with paraformaldehyde and processed for immunodetection of gephyrin as above.

### Fluorescence Image Acquisition and Analysis

Images were acquired with a cooled Micromax CCD camera (Princeton Instruments, Trenton, NJ) mounted onto a Leica (Nussloch, Germany) DRM upright epifluorescence microscope equipped with a 63× objective (NA 1.32) using MetaView software (MetaImaging, Downingtown, PA). Exposure time was determined on highly fluorescent cells to avoid pixel saturation. Quantitations were performed using MetaMorph software (Meta Imaging). A user-defined intensity threshold was applied to select clusters and avoid their coalescence. For quantification of the number and fluorescence intensity of gephyrin and GABA_A_R γ2 clusters, clusters comprising at least 3 pixels were considered.

### Live Cell Staining for Single Particle Imaging

Neurons were stained as described [23]. Briefly, cells were incubated for 5 min at 37°C with primary antibodies against extracellular epitopes of the GABA_A_R γ2 subunit (rabbit: 1∶100; Alomone Labs.; guinea pig: 1∶1000; gift from J.M. Fritschy), the GABA_A_R α5 subunit (guinea pig: 1∶1000; gift from J.M. Fritschy), or the GluA2 subunit of the excitatory AMPA-type receptor (mouse: BD Pharmingen, 1∶100–1∶60), washed, and incubated for 5 min at 37°C with biotinylated Fab secondary antibodies (anti-mouse: 2.5–12 µg/ml; anti-rabbit: 2.5–12 µg/ml; anti-guinea pig: 4–12 µg/ml; Jackson Immunoresearch). After washes, cells were incubated for 1–2 min with streptavidin-coated QDs emitting at 605 nm (1 nM; Invitrogen) in borate buffer (50 mM) supplemented with sucrose (200 mM). Following QD labeling, cultures were exposed for 30 s to the styryl dye *N*-(3-triethylammoniumpropyl)-4-(6-(4-(diethylamino)phenyl)hexatrienyl)pyridinium dibromide (FM 4-64; 2 µM; Invitrogen) and to KCl (40 mM) to stimulate presynaptic vesicle recycling. All washes and incubation steps were performed in imaging medium.

### QD Imaging

Cells were imaged at 37°C in an open chamber mounted onto an Olympus IX71 inverted microscope equipped with a 60× objective (NA 1.45; Olympus, Tokyo, Japan). The fluorescent probes were detected using a Xe lamp and appropriate filter sets (ref in [23]). GFP, FM 4-64 images and QD real time recordings were acquired with a EMCCD camera (Cascade 512B; Roper Scientific, Evry, France) and MetaView software (Meta Imaging). Real time fluorescence images were obtained with an integration time of 75 ms with 360–600 consecutive frames. Cells were imaged within 30 min after FM 4-64 staining. Acid wash assays indicated that GABA_A_Rγ2 and α5 subunits are mostly localized at the cell surface during this recording period (data not shown).

### Single Particle Tracking and Analysis

Single QDs were identified by their blinking property, i.e. their random alternation between emitting and non emitting state [57]. Single QD tracking and reconstruction of trajectories over the recording were performed with homemade software (Matlab; The Mathworks, Natick, MA) as described in [58]. Subtrajectories of single QDs with ≥30 points without blinks were retained. Spots were classified as synaptic when they overlapped or were within 2 pixels (380 nm) from FM 4-64 spots or within 1 pixel (190 nm) from venus-gephyrin, mRFP-gephyrin or GFP-homer1c clusters. Values of the mean square displacement (MSD) plot versus time were calculated for each trajectory by applying the relation: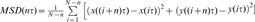 ([59]), where *τ* is the acquisition time, *N* is the total number of frames, *n* and *i* are positive integers with *n* determining the time increment. Diffusion coefficients were calculated by fitting the first four points without origin of the MSD versus time curves with the equation: 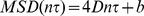
where *b* is a constant reflecting the spot localization accuracy. Trajectories with D<10^−4^ ****µm^2^ s^−1^ were considered immobile and were excluded from the calculation of median D. In our experiments, 3–15% of spots were immobile. For QDs exchanging between synaptic and extrasynaptic compartments, the dwell time inside the synapse, was measured as previously described [31]. Dwell times ≤5 frames (375 ms) were not retained. The explored area of each trajectory was defined as the MSD value of the trajectory for time intervals between 0.3 and 0.375 s [35].

### Fluorescence Recovery After Photobleaching

Experiments were conducted using a FRAP system (FRAP L5D, Roper Scientific, Evry, France) run by MetaMorph software (Meta Imaging Software, Roper Scientific, Evry, France) using routines developed by the Curie Institute Imaging Center (Paris, France). It consist of an inverted microscope (Eclipse TE2000-E; Nikon) equipped with an autofocus system (Nikon), a DG-4 illumination system (Sutter Instruments) and appropriate filter sets (Semrock; Optoprim, Paris, France). Coverslips were mounted on a custom-made chamber and observed with a 100× objective (Nikon; Roper Scientific, Evry, France). Chamber and objective were heated at 36°C. For FRAP, 2–5 circular regions (radius = 0.6 µm) on top of synaptic spots or on extrasynaptic membranes on different neurites of each cell were bleached by high-intensity 488 nm laser (ERROL, Paris, France) for 5 ms at 65 mW, reducing fluorescence by ∼80%. Recovery was monitored by time lapse acquisitions with a CCD camera (QuantEM 512SC, Roper Scientific, Evry, France) at 1 Hz for the first 5s, then at 0.2 Hz for 60s and finally at 0.05 Hz for the subsequent 200 s. Images were analyzed with built-in functions of Metamorph software. Data were normalized and corrected for ongoing photobleaching according to the following equation [34]: *Fcorrt* = (*Ft*/*F0*)/(*Fnbt*/*Fnb0*) where *Ft* is the fluorescence at time *t*, *F0* is the fluorescence before bleaching, *Fnbt* is the average fluorescence intensity of three non-bleached spots at time *t*, and *Fnb0* is the average fluorescence intensity of the same non-bleached spots before bleaching. Best fits of FRAP recovery curves were made according to the following equation [34]: *Ft* = *Pf* [1– (1– *Fbl*) exp (-*t*/*τf*) ] + (1– *Pf*) [1– (1 - *Fbl*) exp (-*t*/*τs*) where *Pf* is the relative size of the fast pool (expressed as a fraction of 1), *Fbl* is the normalized fluorescence immediately after the photobleaching procedure, and *τf* and *τs* are the recovery time constants for the fast and slow pools, respectively.

### Statistical Analyses and Image Preparation

Data were compiled and analyzed using Microsoft Excel (Microsoft, Les Ulis, France). Data are presented as median or mean ± SEM. Means were compared using the nonparametric Student’s t-test. Cumulative distributions were compared using the Kolmogorov-Smirnov (KS) test. Differences were considered significant for P values above 5%. Tests were performed using StatView (SAS, Grégy-sur-Yerres, France). Images were prepared for printing using Photoshop (Adobe Systems, San Jose, CA).

## Supporting Information

Figure S1 **SEP-GABA_A_R γ2 clusters are localized at the neuronal cell surface.** (A) Live cell imaging of recombinant SEP-GABA_A_R γ2 (green) and mRFP-Gephyrin (red) in hippocampal neurons transfected at DIV10. Scale bar, 1 µm. When live cell imaging was done in imaging medium at pH 7.4 (left column), SEP-GABA_A_R γ2 formed numerous fluorescent clusters along the neurite that colocalized with mRFP-Gephyrin fluorescent clusters. After a brief wash at pH 4 (middle), most fluorescence associated with SEP- GABA_A_R γ2 but not with mRFP-Gephyrin was eclipsed. The eclipsed SEP-GABA_A_R γ2 fluorescence rapidly returned in pH 7.4 buffers (right column).
(JPG)

Figure S2 **Diffusion properties of GluA2 in excitatory and inhibitory synapses.** Instantaneous diffusion coefficients (A, C) and MSDs as a function of time (B, D) for GluA2 trajectories exemplified in Fig. 5 A at excitatory synapses (A–B) and at inhibitory synapses (C–D). Color code: red, green and black, QD trajectories at excitatory synapses, inhibitory synapses and at extrasynaptic site, respectively.
(JPG)
