# Supplementary figures and images for: Diffusion Barriers Constrain Receptors at Synapses
Source: PLoS One. 2012 Aug 13;7(8):e43032. doi: 10.1371/journal.pone.0043032 (PMC3418229; doi:10.1371/journal.pone.0043032)

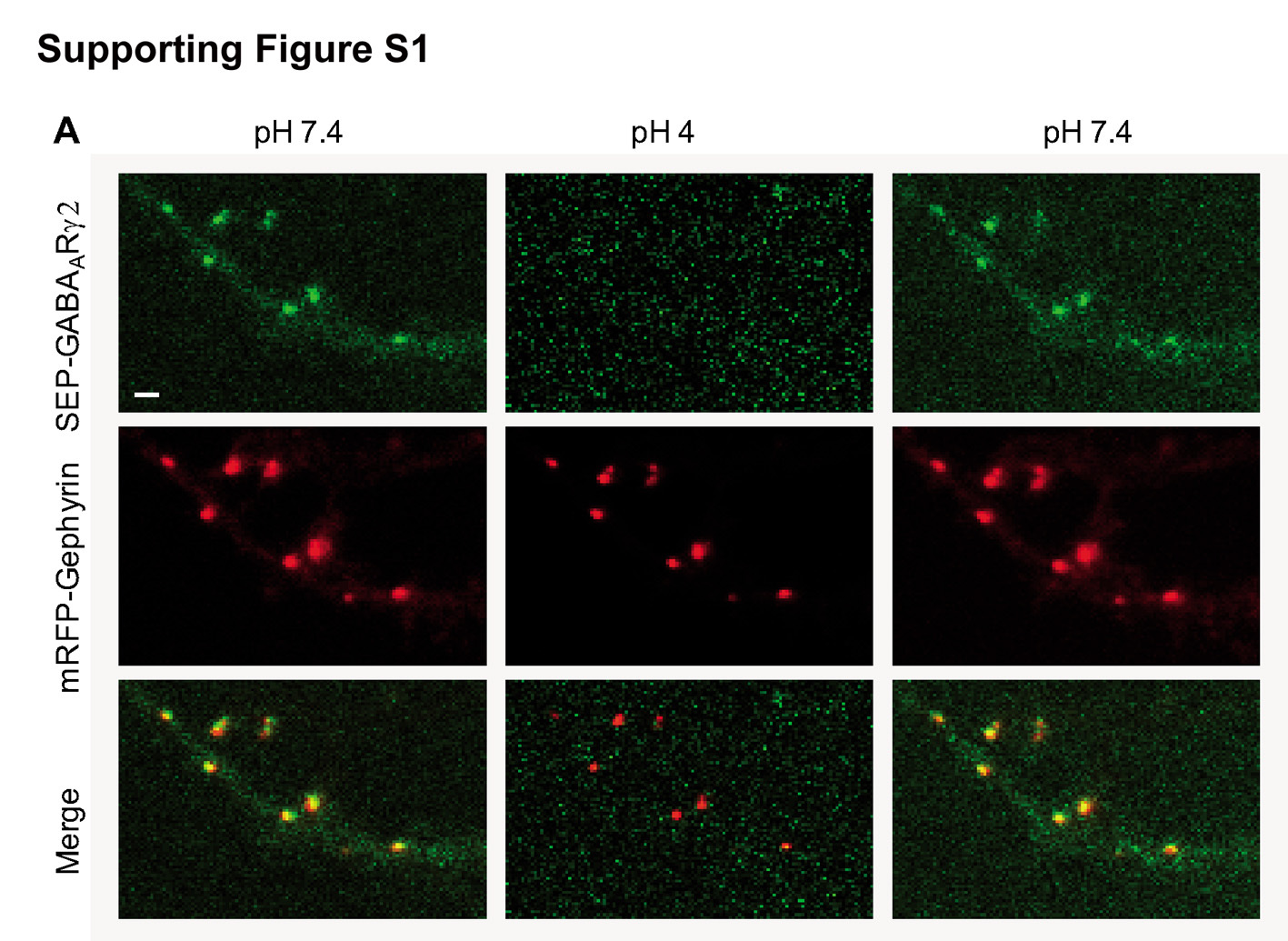

Supplement: Figure S1 — SEP-GABAAR γ2 clusters are localized at the neuronal cell surface. (A) Live cell imaging of recombinant SEP-GABAAR γ2 (green) and mRFP-Gephyrin (red) in hippocampal neurons transfected at DIV10. Scale bar, 1 µm. When live cell imaging was done in imaging medium at pH 7.4 (left column), SEP-GABAAR γ2 formed numerous fluorescent clusters along the neurite that colocalized with mRFP-Gephyrin fluorescent clusters. After a brief wash at pH 4 (middle), most fluorescence associated with SEP- GABAAR γ2 but not with mRFP-Gephyrin was eclipsed. The eclipsed SEP-GABAAR γ2 fluorescence rapidly returned in pH 7.4 buffers (right column). (JPG) [file pone.0043032.s001.jpg]

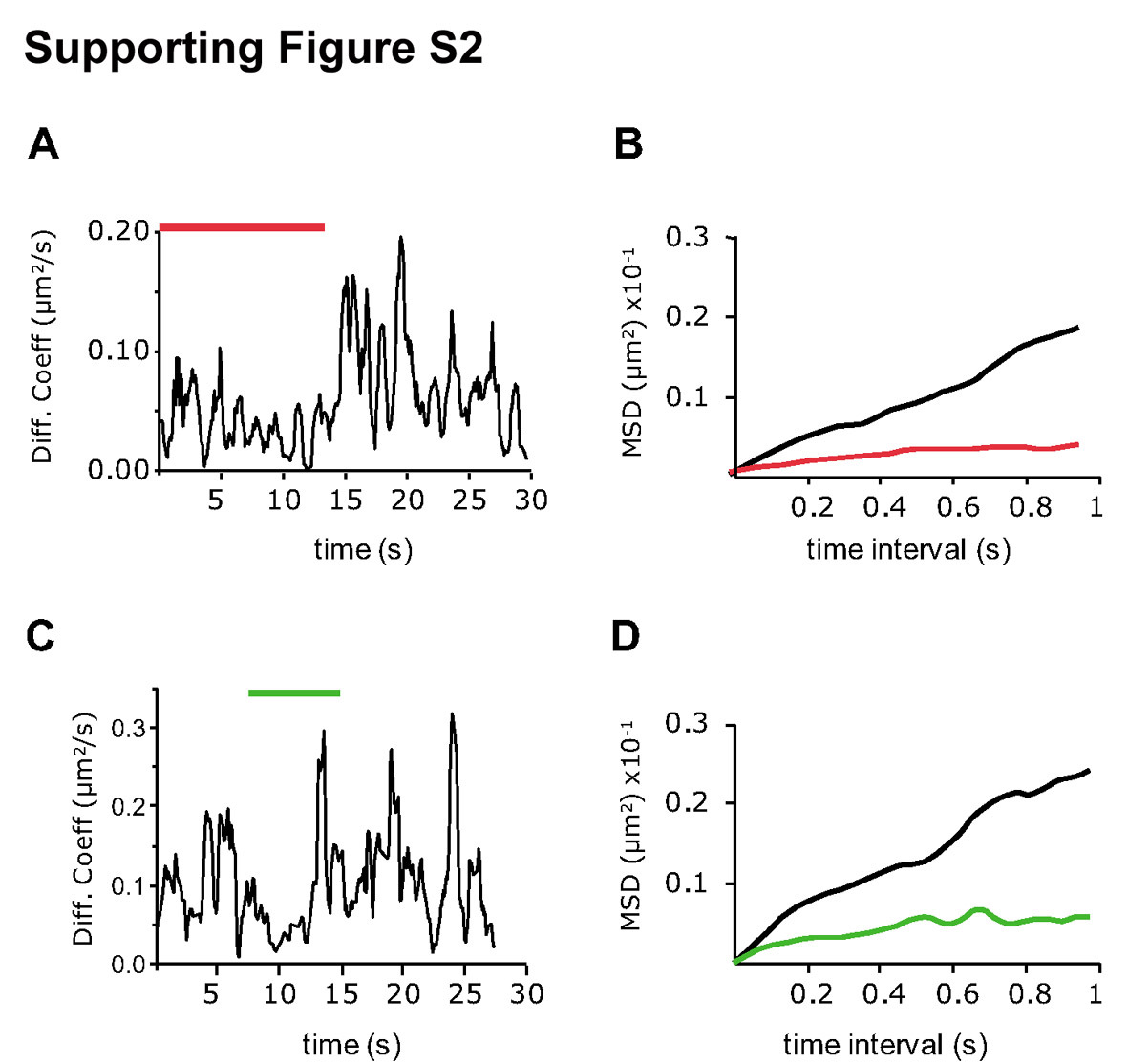

Supplement: Figure S2 — Diffusion properties of GluA2 in excitatory and inhibitory synapses. Instantaneous diffusion coefficients (A, C) and MSDs as a function of time (B, D) for GluA2 trajectories exemplified in Fig. 5 A at excitatory synapses (A–B) and at inhibitory synapses (C–D). Color code: red, green and black, QD trajectories at excitatory synapses, inhibitory synapses and at extrasynaptic site, respectively. (JPG) [file pone.0043032.s002.jpg]
